# Factors associated with dental attendance among adolescents in Santiago, Chile

**DOI:** 10.1186/1472-6831-7-4

**Published:** 2007-04-10

**Authors:** Rodrigo Lopez, Vibeke Baelum

**Affiliations:** 1 Department of Community Oral Health and Pediatric Dentistry, Faculty of Health Sciences, University of Aarhus, Vennelyst Boulevard 9, Aarhus C 8000, Denmark

## Abstract

**Background:**

Dental treatment needs are commonly unmet among adolescents. It is therefore important to clarify the determinants of poor utilization of dental services among adolescents.

**Methods:**

A total of 9,203 Chilean students aged 12–21 years provided information on dental visits, oral health related behavior, perceived oral health status, and socio-demographic determinants. School headmasters provided information on monthly tuition and annual fees. Based on the answers provided, three outcome variables were generated to reflect whether the respondent had visited the dentist during the past year or not; whether the last dental visit was due to symptoms; and whether the responded had ever been to a dentist. Aged adjusted multivariable logistic regression models were used to assess the influence of the covariates gender; oral health related behaviors (self-reported tooth brushing frequency & smoking habits); and measures of social position (annual education expenses; paternal income; and achieved parental education) on each outcome.

**Results:**

Analyses showed that students who had not attended a dentist within the past year were more likely to be male (OR = 1.3); to report infrequent tooth brushing (OR = 1.3); to have a father without income (OR = 1.8); a mother with only primary school education (OR = 1.5); and were also more likely to report a poor oral health status (OR = 2.0), just as they were more likely to attend schools with lower tuition and fees (OR = 1.4). Students who consulted a dentist because of symptoms were more likely to have a father without income (OR = 1.4); to attend schools with low economic entry barriers (OR = 1.4); and they were more likely to report a poor oral health status (OR = 2.9). Students who had never visited a dentist were more likely to report infrequent tooth brushing (OR = 1.9) and to have lower socioeconomic positions independently of the indicator used.

**Conclusion:**

The results demonstrate that socioeconomic and behavioral factors are independently associated with the frequency of and reasons for dental visits in this adolescent population and that self-perceived poor oral health status is strongly associated with infrequent dental visits and symptoms.

## Background

It is widely recognized that adolescents are among the least likely to use the health services [1-4] and some studies have shown that dental care is the most common type of unmet health care need in adolescence [5,6]. It is therefore worrying that adolescents reduce their utilization of dental services [7], and may altogether cease to attend the dentist upon leaving school. This would suggest that emphasis be placed on efforts to secure and reinforce stable dental attendance patterns among children and adolescents. Lack of utilization of dental services is not a random phenomenon, and studies suggest that the dental attendance patterns among the young are related to age [8], gender [8-15], socioeconomic position [9-11,13-17], ethnic background [8,14,15], oral health related behaviors, such as smoking habits [9], and poor self perceived oral health status [14,18]. However, explicit conceptual models have not been presented, and only a single large study comprising a well-defined study group has considered simultaneously the effects of age, gender, and socioeconomic position [13]. This study focused on the frequency of dental visits and selected socioeconomic factors, thus leaving aside possibly important determinants such as the presence of symptoms and the relationship with other oral health related behaviors.

In Chile, the National Health Fund (FONASA) is responsible for providing health care to those 70% of the Chilean population who do not have a private insurance [19-21]. The health care needs of the most affluent 30% of the population are covered by the private insurance system [22]. According to the Chilean legislation, the beneficiaries of FONASA have the right to receive primary dental care free of charge. This dental care includes dental examinations, common intraoral radiographs, emergency treatments, extractions, dental fillings, supragingival scaling and polishing, sealants in permanent molars, topical fluoride, pulpotomies, and endodontic treatment in permanent teeth. Most adolescents are therefore covered by the public oral health-care system.

The aims of this paper were to describe the patterns of dental visits in an adolescent population identified by cluster random sampling; and to explore whether selected socio-demographic and behavioral indicators for oral disease were associated with the dental attendance patterns of adolescents using multivariable models.

## Methods

This analysis was based on data originating from an epidemiological study conducted during year 2000 in Santiago, Chile [23,24]. The local Committee of Ethics of the University of Chile approved the study protocol. The target population comprised all students attending the four grades covering adolescence in the high schools of the Province of Santiago (N ≈ 250,000). This target population represents 85% of the adolescent population of the Province [25]. We used a two-stage random-cluster sampling strategy. Using information on governmental support and the full list of high schools from the Province provided by the Ministry of Education of Chile (N = 618), we generated a list of high schools receiving funds from the public system and another with the private institutions. Each list was permuted at random [26], and lists were then merged to get a random permutation of high schools with publicly funded schools alternating with privately funded schools. The first 133 high schools of the list were contacted to obtain information on the number of students in the last four grades and the number of classes. A total of 104 high schools provided the necessary information and were invited to participate in the study. Six institutions declined to participate, leaving 98 schools in the study.

Second-stage sampling: The size of the schools varied noticeably and a second sampling stage was applied. For schools with few students or fewer than 4 classes, all classes were included in the study. In larger schools, where the number of students in the last four grades was >100 and the number of classes was >3, three classes were randomly selected [23,26]. A total of 310 classes were finally selected and included in the study.

Participation approval was obtained from the headmasters of each selected high school, and informed consent was obtained from the parents of the students. Students were informed about their right to withdraw from the study at any point in time.

A total of 9,203 students aged 12–21 years present in the selected classes were invited to participate and were offered a toothbrush for their participation. All students accepted to fill a brief questionnaire on oral health-related behaviors and conditions [23], while 40 students refused to participate in the clinical examinations. The questionnaire included information on tooth brushing frequency (How often do you brush your teeth? Less than once a day, once a day, more than once a day), smoking habits (Do you smoke cigarettes? No; Yes, sometimes; Yes, daily), their last dental visit (When was the last time you visited a dentist? Less than 6 months ago; 6 to 12 months ago; more than a year ago; never seen a dentist) and the reason for the last dental visit. Why did you visit the dentist?) [23]. The students who received a clinical examination also filled an additional questionnaire on several dimensions of their socioeconomic position. A full description of the questionnaire variables can be found in previous publications [23,27]. Previous analyses showed that some of these indicators were associated with several poor oral health outcomes, including tooth loss, periodontal attachment loss and necrotizing ulcerative gingival lesions [27,28] and these were therefore used in the present analysis. These socioeconomic indicators include the monthly paternal income in thousands of Chilean pesos (no income; <$100; $100–$299; $300–$499; $500–$999; ≥$1000); and the level of education achieved by each of the parents (no education; incomplete primary school; primary school completed; incomplete high school; high school completed; incomplete technical education; technical education completed; incomplete university education; university education completed). The headmasters of the participating schools provided information on the monthly tuition and the annual school fees and this information was used to derive a single 'annual education expense' variable, which was used as an indicator of wealth. Three dichotomous outcome variables were considered. DENTVIS was coded 0 if the student reported to have visited a dentist within the last year, and 1 if this was not the case. SYMPTOM was coded 1 if the student reported that the last dental visits had been due to symptoms, such as pain, bleeding and infection, and coded 0 if the visit was not prompted by the presence of symptoms. NEVER was coded 1 if the student reported to never have visited a dentist, and coded 0 if this was not the case.

Univariable logistic regression analyses were carried out for the three outcomes investigated and the covariates gender; tooth brushing frequency; smoking habits; self-perceived oral health status, and each of the socioeconomic indicators investigated. Variables showing a P-value < 0.25 in the univariable analyses were selected to be included as covariates in age-adjusted multivariable logistic regression analyses for each outcome. The models were built by the consecutive exclusion of one variable from each full model using the likelihood ratio test as described by Hosmer and Lemeshow [29], refitting and verifying the stability of the model after each deletion.

In order to use this modeling approach, only subjects for whom complete data were available for all variables in all models were included in the analyses. At most this led to the exclusion of 11% of the total study population.

Non-significant variables were retained in the models as confounders if their exclusion would result in a change of the estimates by more than 15%. We assessed the interaction between age and gender and the socioeconomic variables included in the models and none was found. It was impossible to assess the presence of other interactions due to collinearity between the variables. Once the final models were built, the goodness-of-fit of each model was evaluated using the Hosmer-Lemeshow goodness-of-fit test using the command 'lfit' in Stata [30]. The option 'robust cluster' for the procedure 'logit' in Stata [30] was used to take into account the fact that the students were nested in classes, which were the ultimate sampling units [23].

## Results

Approximately 93% of the participating students had visited a dentist at least once in their lives, and 43% reported that their last dental visit was more than 1 year before the study. For 7,826 subjects it was possible to classify the reason for visiting the dentist into symptomatic or asymptomatic, and 37% reported symptoms as the reason for their last dental visit. Table 1 shows the distribution of the dental attendance outcomes investigated according to the demographic, behavioral, and socioeconomic characteristics of the study population.

**Table 1:** Distribution of the dental visits outcomes according to the demographic and socioeconomic characteristics of the study population.

Determinant	Dental attendance outcomes (%)		
Overall % in population (100%)	Infrequent visits (n = 8,530)	Never (n = 9,202)	Symptoms related visit (n = 7,826)

Age			
12–14 years (22.5%)	20.4	23.3	19.6
15–17 years (69.5%)	70.9	67.9	70.4
18–21 years (8%)	8.7	8.8	10.0
Gender			
Boy (50.8%)	52.3	51.9	47.1
Girl (49.2%)	47.7	48.1	52.9
Tooth brushing			
More than once a day (70.7%)	68.5	56.4	70.5
Once a day (25.6%)	27.0	37.2	25.8
Less than once a day (3.7%)	4.5	6.4	3.7
Do you smoke cigarettes?			
No (53.2%)	52.2	53.9	49.0
Yes, sometimes (21.8%)	21.3	21.9	23.0
Yes, daily (25%)	26.5	24.2	28.0
Income – father^#^			
≥$500 (26.2%)	19.3	6.7	22.2
$300–$499 (15.3%)	15.4	11.8	14.6
$100–$299 (37.5%)	41.5	44.2	39.7
<$100 (14.0%)	15.8	25.2	15.5
No income (7%)	8.0	12.1	8.0
Education – father^&^			
Technical/university completed (32%)	24.9	11.0	27.8
High school completed (33.9%)	35.5	29.0	34.1
Up to primary school completed (34.1%)	39.6	60.0	38.1
Education – mother^&^			
Technical/university completed (27%)	21.0	9.4	23.5
High school completed (34.7%)	33.2	24.5	33.9
Up to primary school completed (38.3%)	45.8	66.1	42.6
Annual education expenses			
>$760 (24.5%)	17.5	4.0	19.5
$150–$760 (25.5%)	25.1	18.9	24.8
$60–$149 (24.6%)	27.7	37.7	26.7
<$60 (25.5%)	29.7	39.4	29.0
Self-perceived oral health status (Good)			
Good	17.8	16.1	17.4
Regular	68.9	66.9	66.8
Poor	13.3	17.0	15.8

(n) = Number of subjects who provide information for the respective variable.

$ = Thousands of Chilean pesos.

Infrequent visits = Last visit to the dentist was more than a year ago.

Never = Never has visited a dentist.

Symptoms related visit = Last visit to the dentist was due to symptoms.

# = the categories '$500–999' and '≥$1,000' were collapsed into ≥$500.

& = the categories 'no education'; 'incomplete primary school'; 'primary school completed', and 'incomplete high school' were collapsed into 'up to primary school completed'. The categories 'high school completed', 'technical incomplete', and 'university incomplete' were collapsed into 'high school completed'; the categories 'technical education completed' and 'university education completed' were collapsed into 'technical/university completed'.

The logistic regression analyses showed that students who had visited a dentist more than a year ago were more likely to be boys (OR = 1.3); to attend a relatively poor high school (OR = 1.4); to have a father without income (OR = 1.8); to have a father (OR = 1.2), or a mother (OR = 1.5) who only had achieved primary school education; just as they were more likely to report a poor oral health status (OR = 2.0), Table 2.

**Table 2:** Age adjusted logistic regression analysis of socioeconomic determinants of dental attendance patterns among adolescents.

Determinants (reference)	Dental attendance outcomes					
	Infrequent visits		Never		Symptoms related visit	

	OR	[95% CI]	OR	[95% CI]	OR	[95% CI]

Gender (Girl)						
Boy	1.3	[1.1;1.4]	-	-	0.9	[0.8;1.0]
Tooth brushing (>once a day)						
Once a day	1.1	[1.0;1.3]	1.7	[1.4;2.0]	-	-
Less than once a day	1.3	[1.0;1.8]	1.9	[1.3;2.8]	-	-
Do you smoke cigarettes? (No)						
Yes, sometimes	-	-	-	-	1.2	[1.0;1.3]
Yes, daily	-	-	-	-	1.2	[1.0;1.3]
Income – father^# ^(≥$500)						
$300–$499	1.3	[1.1;1.5]	1.6	[1.0;2.4]	1.1	[0.9;1.3]
$100–$299	1.5	[1.3;1.7]	1.6	[1.0;2.4]	1.2	[1.0;1.4]
<$100	1.6	[1.3;2.0]	2.0	[1.3;3.2]	1.2	[1.0;1.5]
No income	1.8	[1.4;2.3]	2.3	[1.5;3.7]	1.4	[1.1;1.7]
Education – father^&^(Techn/univ)						
High school completed	1.2	[1.0;1.4]	1.3	[0.9;1.7]	-	-
Up to primary completed	1.2	[1.0;1.4]	1.6	[1.2;2.2]	-	-
Education – mother^&^(Techn/univ)						
High school completed	1.1	[0.9;1.2]	1.3	[0.9;1.8]	-	-
Up to primary completed	1.5	[1.2;1.7]	1.9	[1.3;2.6]	-	-
Annual education expenses (>$760)						
$150–$760	1.2	[1.1;1.5]	2.9	[1.8;4.9]	1.2	[1.0;1.4]
$60–$149	1.3	[1.1;1.5]	4.0	[2.4;6.8]	1.3	[1.1;1.7]
<$60	1.4	[1.2;1.7]	4.0	[2.3;6.7]	1.4	[1.2;1.7]
Self-perceived oral health status (Good)						
Regular	1.5	[1.3;1.7]	-	-	1.5	[1.3;1.7]
Poor	2.0	[1.6;2.4]	-	-	2.9	[2.4;3.5]

() = Reference category

(Techn/univ) = (Technical or university education completed)

OR = odds ratio

[95% CI] = 95% confidence interval

$ = Thousands of Chilean pesos.

Infrequent visits = Last visit to the dentist was more than a year ago.

Never = Never has visited a dentist.

Symptoms related visit = Last visit to the dentist was due to symptoms.

Subjects who have never visited a dentist were excluded from the model concerning the frequency of dental visits and the model concerning symptoms related visits (Table 2). Subjects who could not be classified in either a symptomatic or an asymptomatic dental attendance pattern were also excluded from the last model.

# = the categories '$500–999' and '≥$1,000' were collapsed into ≥$500.

& = the categories 'no education'; 'incomplete primary school'; 'primary school completed', and 'incomplete high school' were collapsed into 'up to primary school completed'. The categories 'high school completed', 'technical incomplete', and 'university incomplete' were collapsed into 'high school completed'; the categories 'technical education completed' and 'university education completed' were collapsed into 'technical/university completed'.

Students who had never attended a dentist were more likely to have a relatively lower socioeconomic position than those who had visited a dentist at least once, and this finding was consistent whether socioeconomic position was assessed using paternal income, parental achieved education or annual education expenses, just as they were more likely to report that they brush their teeth only once a day (OR = 1.7) or less than once a day (OR = 1.9), (Table 2).

Students who reported that their last visit to the dentist was due to symptoms were more likely to be daily smokers (OR = 1.2), to have a father without income (OR = 1.4); to attend a school with lower tuition and fees (OR = 1.4); and to report a poor oral health status (OR = 2.9), (Table 2).

## Discussion

This study has demonstrated that a substantial proportion of students in this population have never visited a dentist, and that the reason for visiting a dentist is often related to the presence of symptoms. Although it is possible that the reason for never having visited a dentist is to be found in the absence of symptoms, our findings on the socio-economic and oral health behavioral profile of the non-attendees suggest that this interpretation is fallacious. A more plausible explanation for the observations is that economic barriers may prevent a large portion of Chilean adolescents with tangible dental care needs from seeking professional help. Some of the association reported may seem relatively small, however small risks among a large number of people may generate many cases; while a few people with high risk may not do so [31].

The use of self-reported social indicators and self-reported data on dental visits in this study could be seen as a weakness. However, previous studies have shown that adolescents are able to supply valid and reliable information on their parents' socioeconomic position [32-34]; and a study conducted among adults indicate that self-reported data on dental care habits are accurate [35].

The health care needs of socioeconomically deprived Chilean adolescents are thought to be covered by the public system [19-21], and it may seem surprising that dental attendance patterns are strongly associated with social position even in a population that is offered public dental care free of charge for those in the lower end of the social hierarchy. However, this may in part reflect the effect of additional factors such as negative beliefs about dentists [36]; lack of knowledge, and cultural and parental values about the importance of oral health [9,37]; limited access to transportation to and from the health care centers; and the extent to which these health care services are really available. Hence, there are many observations of the public health care system being unable to cope with the current demands for treatment.

Considerably more adolescents in this study population have never visited a dentist or have attended more than one year ago than what has been reported previously for a US American adolescent population [13]. The percentage of subjects who reported their last dental visit being due to symptoms appears to be comparable with that reported for US American and Scottish adolescent populations [9,13].

Our results corroborate previous reports on the role of social inequalities in dental attendance patterns among adolescents [4,9-11,13]. This result strengthens the idea that free access to health care does not guarantee dental care provision among the young, the findings of a study revealing significant ethnic disparities in the utilization of dental services among US American children and adolescents even among Medicaid-eligible subjects points to the same direction [8].

Girls were more likely to visit frequently the dentist than did boys and were more likely to consult the dentist because of symptoms. These results, though not new [8-13], raise questions in connection with the results of previous analyses showing that girls have fewer remaining teeth than do boys in this study population [28]. A plausible explanation for this finding is that visiting a dentist with symptoms increases the probability of tooth extractions because *"the reason for seeking dental treatment care influences the treatment likely to be received" *[38]. This agrees with the results of a previous study showing that children and young adolescents who consulted a dentist because of symptoms were likely to have more teeth missing in later adolescence than were asymptomatic dental attendants, despite a similar number of decayed or filled teeth for both groups at baseline [39].

Our finding of an association between daily smoking and symptom-related dental visits corroborates previous findings on the relationship between smoking and dental attendance patterns [9], but a plausible explanation for the association is missing. It is known that smoking is associated with several other unhealthy behaviors indicating an unhealthy lifestyle [9], and smoking may just be a marker of an environment characterized by health undermining living conditions and an unhealthy lifestyle. This interpretation is corroborated by the association observed between infrequent tooth brushing and infrequent dental visits, and infrequent tooth brushing and having never visited a dentist. Hence, it is well-known that a considerable portion of Chilean adolescents cannot pay for dental visits or even a personal toothbrush [27]. The fact that the students with poor dental attendance patterns were more likely to report their oral health status as poor suggests the existence of substantial unmet dental care needs in this adolescent population.

## Conclusion

The results of the study demonstrate the existence of gender, as well as considerable socioeconomic and behavioral differences in the frequency of, and reasons for dental visits in this adolescent population. Self-perceived oral health status is strongly associated with the frequency of dental visits and the reasons for attendance. The findings of this study indicate that a major impact on dental attendance patterns requires political decisions aiming to reduce social inequalities.

## Competing interests

The author(s) declare that they have no competing interests.

## Authors' contributions

RL conceived and designed the study, collected the data, performed the statistical analysis, the interpretation of the data, and the manuscript drafting.

VB conceived and designed the study, and assisted in the collection, analysis, and interpretation of the data. Both authors reviewed, edited, and approved the manuscript.

## Pre-publication history

The pre-publication history for this paper can be accessed here:


